# Cell-Based Therapies for Cardiac Regeneration: A Comprehensive Review of Past and Ongoing Strategies

**DOI:** 10.3390/ijms19103194

**Published:** 2018-10-16

**Authors:** Andrea Ghiroldi, Marco Piccoli, Federica Cirillo, Michelle M. Monasky, Giuseppe Ciconte, Carlo Pappone, Luigi Anastasia

**Affiliations:** 1 IRCCS Policlinico San Donato, Piazza Malan 2, San Donato Milanese, 20097 Milan, Italy; andrea.ghiroldi@grupposandonato.it (A.G.); marco.piccoli@grupposandonato.it (M.P.); federica.cirillo@grupposandonato.it (F.C.); michelle.monasky@grupposandonato.it (M.M.M.); g.ciconte@gmail.com (G.C.); carlo.pappone@grupposandonato.it (C.P.); 2 Department of Biomedical Sciences for Health, University of Milan, via Luigi Mangiagalli 31, 20133 Milan, Italy

**Keywords:** heart dysfunction, heart failure, cardiac regeneration, stem cells, cell therapy, cardiac clinical trials, translational medicine

## Abstract

Despite considerable improvements in the treatment of cardiovascular diseases, heart failure (HF) still represents one of the leading causes of death worldwide. Poor prognosis is mostly due to the limited regenerative capacity of the adult human heart, which ultimately leads to left ventricular dysfunction. As a consequence, heart transplantation is virtually the only alternative for many patients. Therefore, novel regenerative approaches are extremely needed, and several attempts have been performed to improve HF patients’ clinical conditions by promoting the replacement of the lost cardiomyocytes and by activating cardiac repair. In particular, cell-based therapies have been shown to possess a great potential for cardiac regeneration. Different cell types have been extensively tested in clinical trials, demonstrating consistent safety results. However, heterogeneous efficacy data have been reported, probably because precise end-points still need to be clearly defined. Moreover, the principal mechanism responsible for these beneficial effects seems to be the paracrine release of antiapoptotic and immunomodulatory molecules from the injected cells. This review covers past and state-of-the-art strategies in cell-based heart regeneration, highlighting the advantages, challenges, and limitations of each approach.

## 1. Introduction

Cardiovascular diseases (CVDs) represent one of the leading causes of death in the world. An estimated 17.7 million people died from CVD each year, corresponding approximately to 31% of all global deaths [1]. Acute myocardial infarction (AMI), a life-threatening condition resulting from a complete interruption of the blood flow to the heart [2], is considered one of the major components of CVDs, including nearly 580,000 new coronary attacks and 210,000 recurrent attacks every year in the United States [3]. Nonetheless, AMI induces a characteristic pattern of ultrastructural, cellular, molecular, and metabolic alterations, which leads to irreversible cardiac damage, ultimately culminating in heart failure [4]. In the past 10 years, despite improvements and innovations in CVDs treatment, the number of patients diagnosed with HF increased by 23% [5], thus representing a challenging issue for both the scientific community and the health care systems. The estimated costs associated with HF management have globally reached 108 billion dollars per year [6]. These statistics provide a perfect picture of the magnitude of the impact of chronic heart diseases (CHDs) on society, and the development of novel therapies is urgently warranted.

In the human heart, adult cardiomyocytes (CMs) are able to divide and exhibit spontaneous turnover [7]. Despite this, heart transplantation still represents the gold standard for the treatment of end-stage HF, because it is characterized by an extremely high late-survival rate and by a significant improvement of the patients’ quality of life [8]. Unfortunately, the limited number of heart donors, which is not sufficient to cover the increasing organ demand, and the mandatory transplant-associated lifelong immunosuppressant therapy reduce the feasibility of this therapeutic approach, which could not be applied on a large scale [9]. Moreover, many HF patients are ineligible for heart transplantation because of concomitant comorbidities, and they remain without possible medical, surgical, or interventional treatment options [10]. These are the main reasons why alternative regenerative approaches are extremely needed, and great effort has been directed accordingly by the scientific community for the past 25 years.

The primary therapeutic target of all these innovative strategies is to reduce the myocardial scar by generating new functional cardiac tissue and activating endogenous cardiac-associated mechanisms of repair. The first approach that was attempted was to replace dead cardiomyocytes by the exogenous administration of progenitor cells. Several different cell sources have been employed, ranging from bone marrow stem cells to resident cardiac stem cells [11,12]. More recently, direct cardiac reprogramming strategies have been proposed to generate cardiomyocytes starting from terminally committed cells (i.e., dermal fibroblasts), in order to overcome the technical limitations associated with stem cells [13,14,15]. Moreover, a direct reprogramming approach could be potentially directly applied in vivo on the scar tissue, reverting the cardiac myofibroblasts into cardiomyocytes and restoring the cardiac functionality [16]. However, while several research groups have already demonstrated its efficacy both in vitro [17,18,19] and in animal models [16,20,21], several critical issues still need to be addressed, including the extremely low conversion rate, the incomplete maturation of the induced-cardiomyocytes, and the safety concerns related to the use of genetic material and viruses to induce the transdifferentiation process. Therefore, to date, no clinical applications nor clinical trials have been developed with direct reprogramming approaches [22]. On the contrary, in the past two decades, almost 100 clinical trials with adult stem cells of different tissue origin have been performed [23]. However, while no major adverse safety issues have been observed with the administration of most adult stem cells, the real efficacy of these approaches is still controversial [24].

Indeed, an unbiased comparison of the trial outcomes is challenging, because of the use of stem cells from different tissue sources, as well as the extreme variability in the protocols and in the patient populations that were chosen [25]. Nonetheless, in this review, we tried to provide an accurate analysis of the different cell-based regenerative strategies that have been proposed and developed, illustrating the pros and cons of each approach, drawing an overall picture of where we are today and where we are heading.

## 2. Cell Therapy

### 2.1. Initial Studies with Committed Cells

The first cell therapy attempt (i.e., injection of exogenous cells) for heart regeneration was performed with skeletal myoblasts [26]. This approach was conceived mainly because of the high availability of these cells from autologous sources, their ability to proliferate in vitro and to regenerate skeletal muscle after an injury, and their somewhat high resistance to ischemic insults. Unfortunately, once cells were transplanted into the heart, results were unsuccessful, as they differentiated only into skeletal muscle and not into CMs [27], although some beneficial effects were observed on the infarcted heart of animal models [28,29,30]. Nonetheless, a series of clinical studies using skeletal myoblasts were performed (Table 1) [31,32,33,34]. Early results showed a general amelioration in cardiac function, especially in left ventricular ejection fraction (LVEF), in regional contractility, and in both viability and perfusion of the treated area [35,36], although other clinical studies failed to reproduce these encouraging results [37].

Later, further attempts were made using fetal cardiomyocytes. However, while initial results engrafting fetal CMs in syngeneic recipients seemed promising, as they showed the formation of new myocardium in injured hearts [38,39] and functional improvement after small grafts, long-term studies revealed massive cell death and limited proliferation of the implanted cells, as expected with differentiated cells [40,41]. Therefore, the use of CMs was abandoned, as it was clear that the number of cells needed to regenerate the damaged area had to be incredibly higher than the few cells that survived transplantation. Thus, the scientific community started to look into other cell sources that would be able to proliferate after injection, thus increasing their number, before differentiating [42,43]. Indeed, as described in the next section, stem cells intrinsically possess the ability to self-renew and differentiate into different cell types. For this reason, they have soon become the ideal choice for regenerative heart approaches.

### 2.2. Stem Cells

As stem cell biology has developed over the past two decades, progenitor cells of different sources have become available for regenerative medicine, including cardiac repair. Indeed, several types of stem cells have been investigated, and several therapeutic approaches attempted, as detailed below.

#### 2.2.1. Embryonic Stem Cells

Embryonic Stem Cells (ESCs) have been considered ideal candidates to produce CMs for cardiac repair due to their pluripotency and self-renewal. However, while they were initially isolated in the mouse [44] and other species [45,46,47], they have been available from human sources only relatively recently, as they were isolated from a human blastocyst and cultured in vitro only in 1998 [48]. Successively, CMs were generated from both mouse and human ESCs using different differentiation protocols. The first described method is based on the generation of embryoid bodies, which are spherical cell aggregates formed via self-aggregation of ESCs [49]. The CMs generated from ESCs expressed specific cardiac genes, such as GATA4, Nkx2.5, troponin I, troponin T, α-myosin heavy chain, ventricular myosin light chain, and connexin 43 and 45, proteins typical of gap junctions [50]. The efficiency, which was initially low, was successively increased through a co-culture protocol of ESCs and the endoderm-like cell line, END-2 [51,52], and, successively, by modulating the environment with growth factors to reproduce the in vivo early stage of embryonic cardiac development [53,54]. The first in vivo experiments conducted on rat [55] and pig [50] models showed that ESC-derived CMs were able to proliferate, to express cardiac markers, and to form pacemaker cells, coupling electrically with resident CMs, also after myocardial infarction [56,57,58]. Actually, in a non-human primate model, it has been demonstrated that an intramyocardial delivery of 1 billion human ESC-derived CMs gave re-muscularization of the infarcted zone, despite an incomplete CMs maturation. However, adverse arrhythmic complications were observed [59]. A successive study indicated that the re-muscularization by ESC-derived CMs was able to restore cardiac function after transplantation, as demonstrated by the increase of the global left ventricular ejection fraction after one and three months [60]. In the meantime, the first clinical trial on a human patient was performed showing an improved cardiac functional outcome after three months from the cardiac-committed ESCs implantation [61]. More recently, a small phase I trial (*n* = 6) demonstrated the technical feasibility of producing clinical-grade ESC-derived CMs and their medium-term safety (Table 2) [62].

Overall, while positive results have been reported, the same original concerns about the employment of ESCs on humans are still actual, especially regarding their tumorigenic potential, the immune rejection [63], and the ethical issues on cell isolation from blastocysts.

#### 2.2.2. Adult Stem Cells

Adult stem cells have been isolated from a variety of human tissues. However, their use in cardiac regeneration is limited to the following sources:

● Bone Marrow Stem Cells (BMSCs)

BMSCs are a heterogeneous population of stem cells isolated from the bone marrow that is often divided into two main subtypes, depending on their surface markers: hematopoietic bone marrow stem cells (BM-HSCs) characterized by the expression of CD31, CD34, CD45, and CD133, and mesenchymal bone marrow stem cells (BM-MSCs), which express CD73, CD90, and CD105 [64]. The isolation procedure of the stem cell population from bone marrow is a well-established protocol based on density gradient centrifugation. The resulting product is defined as bone marrow mononuclear cells (BMMNCs), which includes BM-HSCs, BM-MSCs, and committed cells in the various stages of differentiation. BMMNC represent the first stem cell population employed in clinical trials (Table 3), although it has been demonstrated that they do not directly contribute to the formation of the cardiac cell lineage. The TOPCARE-AMI (Transplantation Of Progenitor Cells And Regenerations Enhancement in Acute Myocardial Infarction) trial was one of the first clinical trials with adult stem cells, and it was conducted on 20 randomized patients, receiving an intracoronary infusion of BMMNCs after 4–5 days from an acute myocardial infarction. Results showed a modest increase of the cardiac function after four months of follow-up in addition to the feasibility and safety of the intracoronary infusion of BMMNCs [65]. Many other clinical trials employed BMMNCs, including, for example, the BOOST (BOne marrOw transfer to enhance ST-elevation infarct regeneration) trial [66], the REPAIR-AMI (Reinfusion of Enriched Progenitor cells And Infarct Remodeling in Acute Myocardial Infarction) trial [67], the SWISS-AMI (SWiss multicenter Intracoronary Stem cells Study in Acute Myocardial Infarction) trial [68], or the TIME (Timing In Myocardial infarction Evaluation) trial [69]. The majority of the clinical trials with BMMNCs reported a modest improvement of left ventricular function and cardiac perfusion by enhanced microvascularization [65,66,68,69,70]. Even though the cell population employed was namely the same, there were significant differences among the trials, regarding the number of cells implanted, the delivery method or the patients’ population. These differences made difficult a reliable comparison of the trials’ outcome and also the reproducibility. An example of this aspect is represented by the two versions of the BOOST trial. The former trial was conducted in 2004 on infarcted patients (*n* = 60) who received an infusion of BMMNCs. After six months from the intervention, the treated group showed an increase of 6.7% of the mean global left ventricle ejection fraction, compared to an increase of 0.7% of the control group [66]. However, in the subsequent randomized placebo-controlled, double-blind BOOST-2 trial, they investigated the effects of a low or a high dose of infused cells and the effects of the γ-irradiation, but they failed to reproduce the positive effects observed before [71].

Since BMMNCs include not only stem cells, but also committed cells, several groups conducted clinical trials with a purified population of bone marrow stem cells. In particular, HSCs (CD34^+^ and/or CD133^+^), representing the most abundant stem cell population in BMMNCs (2–4%), were the first purified stem cell population to be used in clinical trials (Table 4). Initially, positive results were obtained by Stamm et al. [78], who described an increase in LVEF and cardiac perfusion six months after transplant. However, in a second study with a more accurate experimental design, they did not reproduce the same results [79]. In this regard, it was demonstrated that HSCs are not able to differentiate into cardiomyocytes once implanted into the heart [80], and that the observed beneficial effects on patients were a consequence of their angiogenic [72,81,82], rather than their differentiation, capacity. Moreover, results from other clinical trials did not show any improvement in cardiac function [69,75,83], highlighting the poor reproducibility of this method, probably due to the different strategies of cell purification, expansion, and concentration [84].

Considering the issues in HSCs manipulation, many groups focused their attention on BM-MSCs, which were easier to use and standardize due to their capacity to be cultured and expanded with well-defined procedures [89], including the potential to differentiate into a variety of adult cell types. Indeed, promising results, characterized by an amelioration of cardiac function and a reduction of the infarct size, were obtained on rodent and swine models [90,91,92]. For these reasons, more than 20 clinical trials were conducted with BM-MSCs (Table 5). In general, the results showed improvements of the cardiac function, demonstrated by an increase of cardiac perfusion or reduction of the infarcted area, accompanied by signs of angiogenesis and reduced fibrosis and scar formation [93,94,95]. However, only a few of these clinical trials presented a well-organized and detailed experimental design and described a measurable improvement of the cardiac function with specific indicators, such as the comparison of the LVEF to a correct control group. Most of these trials observed the beneficial effects at 6 or 12 months of follow-up, while there were no differences between treated and untreated patients at a longer follow-up. However, the enormous effort spent in these trials strongly increased the knowledge of BM-MSCs biology and stem cells therapy. For example, the POSEIDON (PercutaneOus StEm cell Injection Delivery effect On Neomyogenesis) trial demonstrated that BM-MSCs could be used for allogenic transplant without severe immunological response [93], and that to obtain at least little improvements in the clinical outcome, it was necessary to infuse at least 70 million cells, as demonstrated by the SEED-MSC (SafEty and Efficacy of aDult Mesenchymal Stem Cells), C-CURE Cardiopoietic stem Cell therapy in heart failURE), and MSC-HF (Mesenchymal Stem Cells for Heart Failure) trials [96,97,98]. 

● Adipose-Derived MSCs (ADSCs)

ADSCs are mesenchymal stem cells derived from adipose tissue, are more abundant than BMSCs, and have been demonstrated to possess a higher capacity to form colonies than BM-MSCs. Also, they have shown greater expansion potential and more resistance to senescence following the culture passages [114]. On the other hand, the surgical procedure for the isolation of the adipose tissue presents a certain risk of organ injury, sepsis, and pulmonary embolism [115]. The first experiment of ADSCs cardiac infusion on a rat model of chronic myocardial infarction improved the LVEF, preventing wall thinning [116], reducing fibrosis, and promoting angiogenesis [117]. On the contrary, clinical trials in humans showed only modest beneficial effects on cardiac function and on myocardial perfusion [88,118,119,120,121] (Table 6). In particular, a reduction of the infarct size was observed in the APOLLO trial [118] and improvement of the exercise tolerance was described in the ATHENA I/II trial [121]. However, no one revealed a significant improvement in the left ventricle function. Thus, it is still unclear as to what mechanisms are responsible for the effects mediated by ADSCs, also because there is contradictory evidence on their ability to differentiate into cardiomyocytes [122,123]. Most likely, ADSCs exert paracrine effects promoting angiogenesis [124] and secrete various cytokines important for tissue regeneration [125,126].

● Umbilical Cord-Derived MSCs (UC-MSCs)

In the last few years, great interest has been directed to stem cells isolated from a different part of the umbilical cord because of their high capacity of self-renewal and their reduced potential of forming teratomas [127]. Moreover, their isolation is a simple enzymatic digestion protocol performed on medical waste, thus avoiding invasive biopsy or ethical concerns. Among them, cells isolated from the Wharton’s jelly have properties of both ESCs and adult stem cells [128]. Preclinical studies with human cells showed an increase in cardiac vascularization and attenuated remodeling in rat models of myocardial infarction [129,130,131]. Moreover, an increase in cardiac function was also observed in a swine model [132]. The observed improvements were likely the results of a paracrine effect, rather than the differentiation in new cardiomyocytes, as demonstrated by the increase of angiogenesis, the recruitment of endogenous cardiogenic cells, and by the decrease of apoptosis and fibrosis [129,130,131]. Based on these encouraging results, the use of umbilical cord-derived stem cells was also tested in humans to develop a possible new clinical application for cardiac regeneration. The main scope of these trials was first the safety of the allogeneic transplant of the UC-MSCs: interestingly, no severe adverse effects were observed [133,134,135]. Then, four clinical trials included a control group in order to assess a potential therapeutic effect of the UC-MSCs (Table 7). All these trials reported positive results in terms of LVEF in treated patients as compared to controls at different time points (from 6 to 18 months). Moreover, increases in both exercise tolerance and quality of life were observed [136,137,138]. These preliminary results, although promising, need to be confirmed in larger and more organized clinical trials.

● Skeletal Muscle-Derived Stem Cells (Satellite Cells)

Satellite cells are the resident stem cells of the skeletal muscle. These mononucleated myogenic cells proliferate during postnatal growth and their number declines during aging. In the adult skeletal muscle, satellite cells remain quiescent under the basal lamina of the muscle fiber, but they result separated from the fiber itself, and they activate after injury in order to repair muscle damage [140]. In the early 1990s, satellite cells were the first adult stem cell population that were tested for cardiac regeneration in animal models of myocardial injury [141,142]. In particular, the effects of the cardiac injection of canine satellite cells have been evaluated in terms of cells engraftment and differentiation into cardiac-like muscle cells. Histological results confirmed that the cardiac environment was sufficient to induce satellite cells differentiation. However, no functional evaluation has been performed in order to corroborate the actual cardiac recovery after satellite cells transplantation.

Ten years later, a phase I clinical trial has been conducted to examine the feasibility and safety of the intramyocardial transplantation of autologous skeletal muscle-derived satellite cells in patients with non-acute myocardial infarction. Treatment was performed on twelve patients by injecting (100–400) × 10^6^ cells previously expanded in vitro. Results confirmed that the procedure was safe and feasible. Moreover, the satellite cells injection was able to improve the LVEF and the viability of the damaged cardiac tissue (Table 8) [143].

Despite these encouraging results, several critical issues, such as the in vitro expansion procedure and the disability of the differentiated cells to contract simultaneously with the resident cardiac tissue, limited the use of satellite cells for cardiac regeneration strategies [144].

● Cardiac Stem Cells (CSCs)

The ability of regenerating the heart of some animals, including zebrafish [143] and postnatal mice [144], together with some reports of a cell turnover of cardiac cellular components [7], suggested the idea that a population of resident stem cells could be present also in the heart. Indeed, a series of different stem cell populations have been described in mammals, including humans: c-kit positive cells (c-kit+) [145], sca-1 positive cells (sca-1+) [146], cardiosphere-derived cells (CDCs) [147], side population cells [148], isl1 positive cells (isl-1+) [149], and cardiac atrial appendage stem cells (CASCs) [150]. Actually, c-kit+ cells were the first putative CSCs population identified in the heart, which showed promising results after transplantation, attenuating ventricular remodeling and improving cardiac function in mouse and rat models [151,152]. For these reasons, c-kit+ cells were used in the randomized clinical trial SCIPIO (Stem Cell Infusion in Patients with Ischemic cardiomyopathy), demonstrating a significant increase in left ventricular ejection fraction after one year of follow up in patients who received c-kit+ cells injection [153]. However, many independent groups failed to reproduce these positive results, using c-kit+ cells, raising several still-unanswered questions on the real contribution of c-kit+ cells in cardiac regeneration and cardiac function recovery [154,155,156]. Despite this aspect, another clinical trial with c-kit+ cells is ongoing: indeed, the phase II CONCERT-HF (Combination of Mesenchymal and c-kit^+^ Cardiac Stem Cells as Regenerative Therapy for Heart Failure) trial aims to investigate the potential therapeutic effects of the infusion of c-kit+ cells, BM-MSCs or the combination of the two populations on patients with myocardial injury [157]. Another cardiac progenitor cell population has been identified using the stem cell antigen-1 (Sca-1), which is a surface marker of somatic and hematopoietic stem cells. Sca-1+ cells were able to differentiate in beating cardiomyocytes when treated with 5-azacytidine [146] and were shown to reduce ventricular remodeling after transplantation, improving cardiac function by the induction of angiogenesis [146,158]. 

A third type of cardiac progenitors are the cardiosphere-derived cells (CDCs), which have been shown to be clonogenic and have multilineage potential, and that can be safely delivered via intracoronary injections. They have been shown to mediate scar reduction after myocardial infarction, to increase the viable myocardium, and to contribute to the improvement of cardiac function in preclinical models [147]. Recently, CDCs have been used in a clinical trial, CADUCEUS (CArdiosphere-Derived aUtologous stem Cells to reverse ventricular dysfunction), confirming their ability to promote cardiac regeneration, reducing the scar size and thickening the wall of the infarcted zone [159,160,161] (Table 9).

Finally, cardiac atrial appendage stem cells (CASCs) are the most recent cardiac stem cell population isolated. These cells are characterized by high activity of the aldehyde dehydrogenase, and they have shown better cardiomyogenic potential than other CSCs [150]. Preliminary results on a myocardial infarction model in minipig indicated that CASCs could preserve the cardiac function through the cardiomyogenic differentiation of implanted cells [162].

Overall, while all these results with CSCs seem quite promising, the real regenerative potential and, truly, even the existence of some types of CSCs remains controversial, as the reproducibility of some results reported in the literature is under investigation [156]. Moreover, it seems very unlikely that so many different stem cell populations reside inside the cardiac tissue, as compared to other organs, especially considering the poor intrinsic regenerative capacity of the heart itself. At this stage, further unbiased comparative studies are mandatory to fully understand the role of CSCs in ameliorating cardiac function after acute or chronic injuries, analyzing the mechanisms responsible for the reported, yet somehow difficult to be reproduced, beneficial effects.

#### 2.2.3. Induced-Pluripotent Stem Cells (iPSCs)

In 2006, Yamanaka and colleagues developed a method for the generation of ESC-like cells from mouse fibroblasts by the transduction of defined transcription factors (i.e., Oct-3/4, Sox2, Klf4 and c-Myc) through lentiviral vectors [149]. The next year, the same method was used on human fibroblasts to generate human induced pluripotent stem cells (iPSCs) [150], placing a milestone in the field of stem cell biology. These cells, similarly to ESCs, can differentiate into derivatives of all three germ layers both in vitro and in vivo and can form teratomas when implanted in a nude mouse. The differentiation potential of iPSCs has been extensively employed to generate CMs, although the efficiency of the differentiation process was lower than with ESCs [151,152,153]. However, CMs generated from iPSCs showed a pattern of cardiac gene expressions similar to that of hESC-derived CMs, including the expression of Nkx2.5, troponin T, α-myosin heavy chain, α-actinin, ANF, myosin light chain 2 ventricular isoform (MLC2v), and myosin light chain 2 atrial isoform (MLC2a). Moreover, they exhibit spontaneous contractions, fetal-like ion channel patterns [154], and electrophysiological signals [155]. In the past decade, a series of alternative protocols have been developed to improve CMs generation, including the use of bioreactors [156], the application of a two-medium combination culture protocol [157], the implementation of chemical compounds [158,159,160], and the reproduction of the embryonic heart development by modulating the Wnt/β-catenin pathway [161]. At the same time, iPSCs also showed a great potential for clinical use, as demonstrated in several animal models. For example, human iPSCs-derived CMs, cultured on collagen I patches, were able to form grafts with contractile function in adult rat heart [162]. Moreover, iPSCs restored cardiac function and improved left ventricular remodeling in porcine and mice models of myocardial infarction [163], promoting angiogenesis and interstitial networking [164]. To the best of our knowledge, to date, no iPSCs or iPSC-derived CMs transplantation have been performed on humans, as their use in the clinical practice is still premature and unsafe. Although there is little doubt about the regenerative potential of iPSCs, there are still several important limitations that need to be addressed. For example, the efficiency of the reprogramming process is still quite low, and it greatly (and negatively) influences the time necessary to generate a sufficient number of CMs for any possible clinical application (generation of 100–1000 CMs takes at least six months [165]). Moreover, the delivery of the transcription factors used to reprogram the cells was obtained by retroviruses or lentiviruses infections, and this is known to possibly generate genetic mutations or the de novo copy number variations inside the cells genome [166,167]. Finally, iPSCs could have immunogenic properties that could be responsible for adverse immunorejection after cell transplantation [168]. It is clear that a more accurate evaluation of the genetic and epigenetic modifications, as well as the determination of the actual immunogenicity of iPSCs are all necessary before any possible use of these cells in therapeutic strategies. 

## 3. Considerations on Cell Therapy

More than 20 years have passed since the first experiment on cell transplantation for cardiac regeneration [142] was performed (Table 1). Since then, various clinical trials have been conceived and started. However, it is difficult to draw conclusions, mainly because most trials are still in the early phases (I and II), and they can only offer limited, yet essential, information about safety, but only little insight about the efficacy of the approaches. In fact, they still lack the crucial phase III, which is the one step that can tell us about the efficacy of the therapy. Indeed, most of the endpoints selected for the ongoing clinical trials evaluation often have a low therapeutic impact and are not validated and accepted surrogates for the clinical outcome by the major regulatory agencies, such as the U.S. Food and Drug Administration [169]. Indeed, left ventricular ejection fraction, maximum oxygen consumption, brain natriuretic peptide, or myocardial perfusion are not considered hard endpoints, whereas, on the contrary, primary endpoint of phase III clinical trials should reflect clinically relevant effects, such as mortality, readmission, reintervention, defibrillator event, left ventricular assist device placement, or recurrent heart infarct symptoms [170]. Moreover, the majority of cardiac cell-based trials enrolled patients with acute or convalescent MI, who received prompt and optimal percutaneous reperfusion therapies to preserve cardiac function, leaving little space for any consistent improvement. This patient population has low mortality and morbidity, even without any adjunctive cell therapy [169]. A more detailed and accurate process for patients’ selection based on risk stratification would be helpful to determine the responsiveness to the therapy. Therefore, it seems clear that more appropriately targeted trials, in patients with more severe cardiac dysfunction, are needed to establish the real efficacy of cardiac cell therapy as an effective approach for cardiac regeneration and functional repair. In particular, there is a need for their standardization in order to allow a useful comparison of the results and to increase their reproducibility [171]. 

Beyond these concerns about the selection of the endpoints and patient population, there are also technical limitations for the procedures that must be faced [172]. For instance, it is important to define the maturation status of the engrafted cells, because while it has been demonstrated that adult mature CMs do not survive transplantation [39], partially differentiated cells, such as those derived from ESCs or MSCs, can cause arrhythmias [59] or tumor formation [173], negatively influencing the outcome of the intervention. Moreover, and possibly most importantly, there is increasing evidence that most of the injected cells do not survive after transplantation, because they are either immunologically rejected, they do not find a proper microenvironment, they are trapped in pulmonary vasculature, or they diffuse throughout the entire body with the circulatory system [174]. To partially circumvent these difficulties, different routes of cell administration have been tested [175], including surgical intramyocardial injection, catheter-based intramyocardial administration, trans-endocardial injection, trans-coronary venous injection, intravenous infusion, intracoronary artery administration, retrograde coronary venous delivery system, and engineered monolayer tissue transplantation. Overall, each different protocol showed both advantages and disadvantages, and there are no real unbiased and comprehensive comparative studies, suggesting that it is almost impossible to define a standard protocol for cell administration [176]. For example, intramyocardial delivery resulted in better cardiac cell retention and allowed the delivery of a high number of cells. However, this technique is very invasive, and the cell distribution might be even too localized. In contrast, intracoronary delivery is simpler than intramyocardial injection, and it enables a homogeneous distribution inside large myocardial regions. However, cell retention is low and the number of cells that can be delivered by each infusion is limited [177,178,179]. Moreover, it is unclear whether the delivered cells can couple and contract with existing cardiac tissue. In fact, the improvement of cardiac function observed in the clinical trials could be related to other aspects, including paracrine effects due to the secretion of soluble factors by transplanted cells that eventually induce different processes, such as myocardial protection, the activation or amplification of endogenous repair processes, neovascularization, and cardiac remodeling [180,181]. This hypothesis was confirmed by a series of studies, both in vitro and in vivo, on the effects of stem cell exosomes’ treatment on cell protection and cardiac regeneration. The results showed that exosomes increased the apoptosis resistance of cultured cardiomyocytes (Xiao et al., 2016), and, moreover, it augmented the cardiac function of infarcted hearts in animal models [182,183,184,185]. In particular, they induced angiogenesis and cardiomyocyte survival, reducing fibrosis and the left ventricle remodeling. The promising results suggest that exosomes could represent the first step for a cell-free therapy for cardiovascular disease, which would be more advantageous than cell therapy, considering different aspects, such as the costs, the standardization of the therapy, the scale-up of the production process and the possibility to engineer ad hoc.

In conclusion, cell therapies for cardiac regeneration could be valid approaches that still need more accurate and extensive studies to determine and improve their feasibility and efficacy. The long-term therapeutic effects have yet to be monitored in order to determine whether cell therapy has the potential to increase lifespan, decrease mortality, and to improve patients’ quality of life. Moreover, as there are some safety issues, especially with the use of more undifferentiated progenitors, a careful evaluation of the potential side effects of cardiac cell therapy has to be conducted before any clinical application can be foreseen. 

## 4. Conclusions

**Figure 1:**
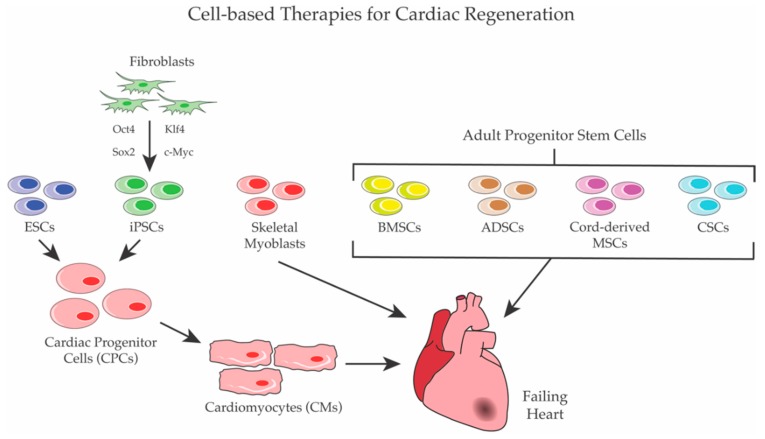
Cell-based Therapies for cardiac regeneration. Several populations of stem cells were used for cardiac regeneration. ESCs and iPSCs were first differentiated to CPCs and/or to CMs before transplantation, whereas skeletal myoblasts and adult stem cells—such as BMSCs, ADSCs, cord-derived MSCs, and CSCs—were directly employed to regenerate heart.

Identifying new therapies for heart diseases, which still represent the primary cause of death in the Western world, is a difficult challenge for modern medicine. As described in this review, in the last two decades, an impressive number of cell-based clinical trials have been conducted with the same overall objective of replacing dead (or damaged) cardiomyocytes with functional ones and restoring the cardiac functionality (Figure 1). However, while the pre-clinical results on animal models generated high expectations, human trials gave very controversial results. In fact, the extremely heterogeneous experimental conditions of the trials, including differences in the cell types used, in the doses and the timings of intervention, in the delivery strategies, in the patients’ selection, and in the time points evaluated, gave rise to often contradictory results. Furthermore, to date, even the most favorable approaches showed only modest long-term outcomes. However, these studies and clinical trials enabled the discovery of critical signaling pathways and transcription-factor networks involved in heart development and regeneration, including cardiac stem cell differentiation. Moreover, it is now unequivocally demonstrated that: (a) cell therapy for cardiac regeneration is feasible and generally safe; (b) the exogenous delivery of adult stem cells suffers from poor engraftment and retention in the heart; and (c) the positive effects of stem cell approaches for cardiac regeneration are mainly mediated by paracrine effects. Therefore, while new cell-based approaches are still under development, many argue that the delivery of regenerating factors, including small molecules and recombinant proteins, might be the winning strategy. Clearly, future trials will need to be closely monitored using more standardized and reliable analytical tools to evaluate the outcomes in order to avoid the previous chaotic generation of unreproducible data, keeping in mind that the number of cardiac diseases are increasingly growing, and patients are in urgent need of novel therapies. 

## Figures and Tables

**Table 1:** Clinical trials with committed cells (skeletal myoblasts) for cardiac regeneration.

Reference	Clinical Trial	Disease	Delivery Method	Subjects	LVEF Improvement
Pagani [32]	Phase I	ICM	SI	Treated: 5	Not shown
Siminiak [33]	Phase I	AMI	SI	Treated: 10	Yes
Menasche [37]	MAGIC	CHF	SI	Treated: 97 Control: 30	Not shown
Povsic [34]	MARVEL-I	CHF	SI	Treated: 15 Control: 8	Not shown

ICM: ischemic cardiomyopathy; SI: sub-epicardial injection; AMI: acute myocardial infarction; CHF: chronic heart failure.

**Table 2:** Clinical trials with ESCs for cardiac regeneration.

Reference	Clinical Trial	Disease	Delivery Method	Subjects	LVEF Improvement
Menasche [61]	Case Report	HF	FS	1	Yes
Menasche [62]	Phase I	ICM	FS	Treated: 6	Not shown

HF: heart failure; FS: fibrin scaffold; ICM: ischemic cardiomyopathy.

**Table 3:** Clinical trials with BMMNCs for cardiac regeneration.

Reference	Clinical Trial	Disease	Delivery Method	Subjects	LVEF Improvement
Hamano [72]	Phase I	ICM	IM	Treated: 5	Not shown
Strauer [70]	Phase I	AMI	IC	Treated: 1 0Control: 10	Yes
Assmus [65] Leistner [73]	TOPCARE-AMI	AMI	IC	Treated: 59	Yes
Wollert [66]	BOOST	AMI	IC	Treated: 3 0Control: 30	Yes
Lunde [74]	ASTAMI	AMI	IC	Treated: 24 Control: 25	Not shown
Schachinger [67]	REPAIR-AMI	AMI	IC	Treated: 101 Control: 103	Yes
Surder [68] Suerder [75]	SWISS-AMI	AMI	IC	Treated: 128 Control: 64	No
Wohrle [76]	SCAMY	AMI	IC	Treated: 29 Control: 13	No
Strauer [77]	STAR-heart	ICM	IC	Treated: 191 Control: 200	Yes
Traverse [69]	The TIME Study	AMI	IC	Treated: 79 Control: 41	No

ICM: ischemic cardiomyopathy; IM: intramyocardial injection; AMI: acute myocardial infarction; IC: intracoronary infusion.

**Table 4:** Clinical trials with BM-HSCs for cardiac regeneration.

Reference	Clinical Trial	Disease	Delivery Method	Subjects	LVEF Improvement
Stamm [78]	Phase I	ICM	IM	Treated: 35 Control: 20	Yes
Tendera [85]	REGENT	AMI	IC	Treated: 16 0Control: 40	No
Povsic [86]	RENEW	RA	IM	Treated: 57 Control: 55	Not shown
Noiseux [87]	IMPACT-CABG	ICM	IM	Treated: 2 0Control: 20	Not shown
Quyyum [88]	PreSERVE-AMI	AMI	IC	Treated: 78 Control: 83	Yes

ICM: ischemic cardiomyopathy; IM: intramyocardial injection; AMI: acute myocardial infarction; IC: intracoronary infusion; RA: refractory angina.

**Table 5:** Clinical trials with BM-MSCs for cardiac regeneration

Reference	Clinical Trial	Disease	Delivery Method	Subjects	LVEF Improvement
Chen [99]	Phase II	AMI	IC	Treated: 34 Control: 35	Yes
Chen [100]	Phase II	AMI	IC	Treated: 24 Control: 24	No
Hare [101]	Phase I	AMI	IV	Treated: 39 Control: 21	No
Yang [102]	Phase I	AMI	IC	Treated: 16	No control
Hare [93]	POSEIDON	ICM	IM	Treated: 31	No control
Bartunek [96]	C-CURE	ICM	IM	Treated: 32 Control: 15	Yes
Gao [103]	Phase II	AMI	IC	Treated: 21 Control: 22	No
Rodrigo [104]	Phase I	AMI	IM	Treated: 9 Control: 45	No
Karantalis [105]	PROMETHEUS	ICM	IM	Treated: 6	No control
Heldman [94]	TAC-HFT	ICM	IM	Treated: 22 Control: 11	No
Lee [97]	SEED-MSC	AMI	IC	Treated: 33 Control: 36	Yes
Ascheim [106]	Phase II	ICM	IM	Treated: 2 0Control: 10	No
Chullikana [107]	Phase I/II	AMI	IV	Treated: 1 0Control: 10	No
Perin [108]	Phase II	ICM	IM	Treated: 45 Control: 15	No
Mathiasen [98]	MSC-HF	ICM	IM	Treated: 4 0Control: 20	Yes
Guijarro [109]	MESAMI	ICM	IM	Treated: 10	No control
Xiao [110]	-	DC	IC	Treated: 17 Control: 20	Yes
Florea [111]	TRIDENT	ICM	IM	Treated: 30	No control
Butler [112]	Phase II	Non-ICM	IV	Treated: 11 Control: 12	No
Bartunek [113]	CHART-I	ICM	IM	Treated: 12 0Control: 151	No

AMI: acute myocardial infarction; IC: intracoronary infusion; IV: intravenous injection; ICM: ischemic cardiomyopathy; IM: intramyocardial injection; DC: deleted cardiomyopathy; non-ICM: non-ischemic cardiomyopathy.

**Table 6:** Clinical trials with ADSCs for cardiac regeneration.

Ref.	Clinical Trial	Disease	Delivery Method	Subjects	LVEF Improvement
Houtgraaf [118]	APOLLO	AMI	IC	Treated: 1 0Control: 4	No
Perin [119]	PRECISE	ICM	IM	Treated: 21 Control: 6	No
Henry [121]	ATHENA I	ICM	IM	Treated: 17 Control: 14	No
Kastrup [120]	ATHENA II	ICM	IM	Treated: 10	No control
Qayyum [88]	MyStromalCell	ICM	IM	Treated: 41 Control: 20	Not shown

AMI: acute myocardial infarction; IC: intracoronary infusion; ICM: ischemic cardiomyopathy; IM: intramyocardial injection.

**Table 7:** Clinical trials with UC-MSCs for cardiac regeneration.

Reference	Clinical Trial	Disease	Delivery Method	Subjects	LVEF Improvement
Li [134]	-	ICM	IC	Treated: 15	No control
Musialek [135]	-	AMI	IC	Treated: 10	No control
Fang [133]	-	ICM	IV	Treated: 3	No control
Zhao [138]	-	ICM	IM	Treated: 3 0Control: 29	Yes
Gao [137]	Phase II	AMI	IC	Treated: 58 Control: 58	Yes
Can [139]	HUC-HEART	ICM	IM	Treated: 18 Control: 4	Not shown
Bartolucci [136]	RIMECARD	ICM	IV	Treated: 15 Control: 15	Yes

ICM: ischemic cardiomyopathy; IC: intracoronary infusion; AMI: acute myocardial infarction; IV: intravenous injection; IM: intramyocardial injection.

**Table 8:** Clinical trial with satellite cells for cardiac regeneration.

Reference	Clinical Trial	Disease	Delivery Method	Subjects	LVEF Improvement
Herreros [143]	Phase 1	Non-AMI	SI	Treated: 12	Yes

Non-AMI: non-acute myocardial infarction; SI: sub-epicardial injection.

**Table 9:** Clinical trials with CSCs for cardiac regeneration.

Reference	Clinical Trial	Disease	Delivery Method	Subjects	LVEF Improvement
Bolli [145]	SCIPIO	ICM	IC	Treated: 16 Control: 5	Yes
Makkar [146] Malliaras [147]	CADUCEUS	AMI	IC	Treated: 17 Control: 8	Yes
Bolli [148]	CONCERT-HF	ICM	IM	Treated: 9 Control: 9	Not shown

ICM: ischemic cardiomyopathy; IC: intracoronary infusion; AMI: acute myocardial infarction; IM: intramyocardial injection.
